# Case–Control Study of Factors Associated with Hemolytic Uremic Syndrome among Shiga Toxin–Producing *Escherichia coli* Patients, Ireland, 2017–2020

**DOI:** 10.3201/eid3104.240060

**Published:** 2025-04

**Authors:** Diana Espadinha, Melissa Brady, Carina Brehony, Douglas Hamilton, Lois O’Connor, Robert Cunney, Suzanne Cotter, Anne Carroll, Patricia Garvey, Eleanor McNamara

**Affiliations:** European Programme for Public Health Microbiology Training, European Centre for Disease Prevention and Control, Solna, Sweden (D. Espadinha); National Reference Laboratory for STEC at Public Health Laboratory Health Service Executive, Cherry Orchard Hospital, Dublin, Ireland (D. Espadinha, A. Carroll, E. McNamara); European Programme for Intervention Epidemiology Training, European Centre for Disease Prevention and Control, Solna (M. Brady); Health Service Executive Health Protection Surveillance Centre, Dublin (M. Brady, C. Brehony, S. Cotter, P. Garvey); Health Service Executive National Social Inclusion Office, Dublin (D. Hamilton); Health Service Executive Public Health, Dr. Steevens’ Hospital, Dublin (L. O'Connor); Children's Health Ireland at Temple Street, Dublin (R. Cunney); Royal College of Surgeons in Ireland, Dublin (R. Cunney); Trinity College Dublin School of Medicine and Saint James's Hospital, Dublin (E. McNamara)

**Keywords:** Shiga toxin–producing Escherichia coli, STEC, hemolytic uremic syndrome, HUS, bacteria, antimicrobial resistance, enteric infections, food safety, Ireland

## Abstract

Shiga toxin–producing *Escherichia coli* (STEC) infection can cause potentially fatal hemolytic uremic syndrome (HUS). To determine epidemiologic and bacterial genomic factors associated with HUS, we conducted a retrospective case–control study with 108 HUS cases and 416 unmatched controls (non-HUS) selected among STEC notifications in Ireland during 2017–2020. We combined routinely collected epidemiologic data on STEC notifications with genomewide association study findings and used logistic regression to estimate adjusted odds ratios. Our findings reaffirmed known risk factors, such as young age (0–9 years) and presence of specific *stx* genes or gene combinations (*stx*2*a*; *stx1a* + *stx2a*; *stx1a* + *stx2c*), and additionally suggest that having outbreak-associated infection, residence within the East region of Ireland, and the combined presence of both *ygiW* and group_5720 or both *pfkA* and *fieF* genes are potentially associated with developing HUS. Our findings could improve early identification of high-risk STEC infections and help guide enhanced surveillance and public health management.

Shiga toxin–producing *Escherichia coli* (STEC) are a major cause of gastroenteritis worldwide. Transmission routes include person-to-person spread, animal contact, ingestion of untreated water, and consumption of contaminated food, including minced beef products and fresh produce such as lettuce and spinach (*1*). Symptoms range in severity from diarrhea and bloody diarrhea to the potentially fatal condition hemolytic uremic syndrome (HUS), which is characterized by microangiopathic hemolytic anemia, thrombocytopenia, and acute kidney injury (*2*). A combination of host, environmental, and bacterial factors have been identified as contributors to HUS, including young age, bloody diarrhea and vomiting, antimicrobial drug treatment, and presence of specific Shiga toxin *stx* genes, the intimin *eae* gene, and the entero-hemolysin *ehxA* and α-hemolysin *hlyA* genes (*3**–**6*).

STEC has long been a public health problem in Ireland, which has reported the highest incidence rate among European Union Member States for many years; in 2018, the crude rate was 20.0 cases/100,000 population, nearly 10 times the average for Europe (*7*). In 2017, a total of 2.9% (n = 27) of reported STEC cases in Ireland led to HUS (*1*).

Despite past research and increased availability of microbial genomic information resulting from a rise in the application of molecular-based approaches to diagnose STEC infections (*8*), identification of factors that place patients at higher risk of HUS remains difficult. To gain new insights into factors potentially associated with HUS, we conducted a case–control study linking epidemiologic data reported on Ireland’s Computerised Infectious Disease Reporting (CIDR) system to complete pathogen molecular characterization data. Our investigation included a genomewide association study (GWAS) to identify novel genes associated with HUS in STEC isolate genomes.

## Methods

### Study Design and Record Linkage

In this retrospective case–control study, we selected patients from a national cohort of 3,735 persons notified as having STEC infection to Ireland’s Health Protection Surveillance Centre via CIDR during January 1, 2017–December 31, 2020. We linked epidemiologic and laboratory data from CIDR to laboratory records from the National Reference Laboratory for STEC at the Public Health Laboratory HSE Dublin. In total, 3,486 (93%) CIDR notifications could be linked to a laboratory record, 1,457 (39%) by using laboratory specimen identification and 2,029 (54%) by using a combination of variables (date of birth, sex, county of residence, specimen collection date, and report date). We validated linkage with Regional Departments of Public Health, which have responsibility for notifying STEC infections and related HUS and STEC outbreaks, according to a standard surveillance case definition (*9*). In line with the surveillance definitions of the European Union, we defined an HUS patient as an STEC patient who had acute renal failure and microangiopathic hemolytic anemia, thrombocytopenia, or both (*10*).

Whole-genome sequencing (WGS) results were available for 2,911 (84%) linked records. We selected patients from among those that met the inclusion criteria (n = 2,296 [66%]): having available WGS data and either having a sporadic infection (not outbreak associated) or being part of an outbreak. Only 1 patient from each outbreak was included, to mitigate potential bias from including the same strain multiple times and because of the lower threshold for testing during outbreak investigations. Case-patients were those who were notified as having STEC infection and who had related HUS. Controls were defined as patients who were notified as having STEC infection but who did not have HUS. Patients who had a clinical diagnosis but no laboratory sample could not be included.

### Sample Size Estimation

We applied Fleiss formulas for unmatched case–control studies with continuity correction to estimate the minimum sample size for case-patients (n = 16) and controls (n = 64), given the power 0.8, signiﬁcance level of p = 0.05, case-control ratio of 1:4, and target odds ratio (OR) of >2.0. We determined the probability of exposure (0.9 in case-patients and 0.5 in controls) on the basis of results of *stx2* in a multivariable analysis of risk factors for STEC-related HUS conducted by other researchers (*11*). The final sample size was 514 patients, comprising all 108 cases that met the inclusion criteria (representing 82% of STEC patients who had HUS develop during the study period) and 416 unmatched controls.

### Variables

We included epidemiologic variables routinely collected by standardized questionnaire (*12*). Those categories were age (categorized as 0–9 years or >10 years), sex, notification date, residence status in Ireland, public health administrative region (within Ireland) (*13*), outbreak association, reported vomiting, reported bloody diarrhea, residence in an urban or rural location (urban location defined as a settlement of >1,500 people), travel abroad within 10 days before illness onset, type of home drinking water (public or private), reported consumption of unpasteurized cheese or milk in the 10 days before illness onset, risk group (child attending crèche, childcare worker, or food handler), recent (within 10 days before illness onset) outdoor activities or recreational farmland contact (hillwalking, camping, swimming in lakes, water sports, or going to a beach), contact with farm animals or their feces, and HUS. An outbreak was defined as the occurrence of >2 cases that shared an epidemiologic link (a potential common source) or where the observed number of cases exceeded the expected number. We extracted the following genomic variables from isolates recovered from patients by the NRL: serogroup, *stx* genes or subtypes, *eae* genes or subtypes, *ehxA* gene, and genes with significant associations with HUS in the GWAS (Appendix 1).

### WGS

The study dataset included genomes of 531 STEC isolates from 524 patients. All microbial culture and PCR testing at the NRL was ISO 15189 accredited. We excluded isolates from repeated sampling of the same patient (within the same episode of infection) unless the serogroup was different. We considered an episode of infection resolved if a patient had 2 negative stool samples 48 hours apart. Seven patients had isolates from 2 different episodes of infection; we included isolates from both episodes in the analysis. 

The distribution of isolates by year was as follows: 2017, n = 99; 2018, n = 154; 2019, n = 135; and 2020, n = 143. Isolates collected in 2017 were sequenced at the UK Health Security Agency Gastrointestinal Bacteria Reference Unit. From 2018 onward, all isolates were sequenced at the NRL. In brief, bacterial genomic DNA was extracted using a MagNA Pure 96 automated station (Roche Diagnostics, https://www.roche.com), according to the manufacturer’s instructions. DNA library preparation was performed using Nextera chemistry and MiSeq platform for sequencing (paired-end reads, read length 300 bp) (Illumina, https://www.illumina.com). The paired-ended reads were imported into BioNumerics version 8.1 (bioMérieux, https://www.biomerieux.com) and quality control and trimming performed according to default settings, and genomes assembled de novo with SPAdes (https://github.com/ablab/spades).

### In Silico Virulence and Serogroup Analysis

Serogroup, *stx* subtype, and presence of *eae* and *ehxA* genes were detected through BioNumerics’ built-in search functions. The *eae* gene subtypes were determined using a BLAST search (https://blast.ncbi.nlm.nih.gov) of a manually curated in-house database established in the BioNumerics platform by collecting the nucleotide sequences of *eae* subtypes described in the literature (*14*–*16*).

### Pangenome and Genomewide Association Studies

We performed further bioinformatic analyses by using tools available on the Galaxy Europe Server platform (https://usegalaxy.eu) (*17*). We annotated draft genomes by using Prokka Galaxy Version 1.14.6+galaxy1 (*18*) with the *E. coli* genus BLAST database. We then used Roary Galaxy version 3.13.+galaxy2 (*19*) in the pangenome creation, with loci defined by alleles with a minimum of 95% blastp identity and split paralogs enabled. We defined core genes as genes present in >99% of the genomes, the remaining genes were defined as accessory. We used Scoary Galaxy version 1.6.16+galaxy0 (*20*) to determine significant associations between accessory genes and HUS status. To control the false discovery rate associated with multiple comparisons, we considered genes positively associated if the OR was >1 and the Benjamini-Hochberg p value <0.05. We used pairwise comparisons with p<0.05 as a threshold to minimize the lineage confounding effect. We explored the putative function of genes annotated as hypothetical proteins by performing a BLAST search of consensus sequence against other databases such as UniProt (*21*) and STRING (*22*).

### Phylogenomic Analysis

We generated a maximum-likelihood tree by using RaxML (*23*) on the basis of a multi-FASTA alignment of the core genes of the 531 STEC isolates (Figure). We annotated and visualized the final tree by using iTOL version 6.8.1 (https://itol.embl.de) (*24*).

**Figure:**
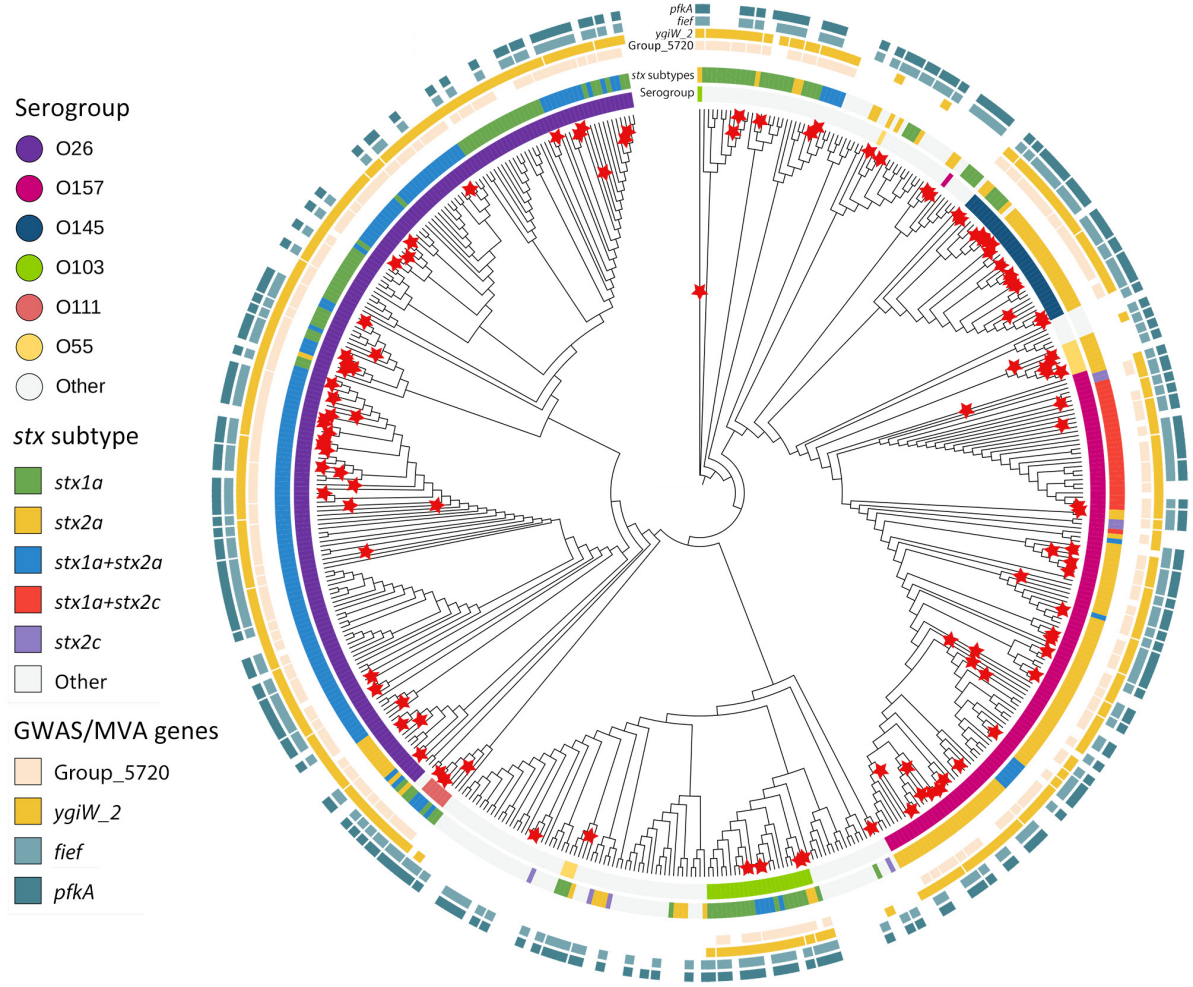
Maximum-likelihood phylogenetic tree of HUS and non-HUS STEC isolates from study of HUS among patients with STEC, Ireland, 2017–2020. Tree was generated by using RaxML (*23*) on the basis of a multi-FASTA alignment of the core genes of the 531 STEC isolates. We annotated and visualized the final tree by using iTOL version 6.8.1 (https://itol.embl.de) (*24*). HUS cases (indicated by red stars) were distributed across several serogroups: O26 (36%), O157 (26%), O145 (14%), O103 (4.6%), O111 (2.8%), and O55 (5.6%). GWAS, genomewide association study; HUS, hemolytic uremic syndrome; MVA, multivariable logistic regression analysis; STEC, Shiga toxin–producing *Escherichia coli*.

### Statistical Analyses

We performed statistical analyses by using the *glm* function in R version 4.0.3 (The R Project for Statistical Computing, https://www.r-project.org) and the *car* (*26*) and generalhoslem (*27*) packages. We first explored the relationship between case-patients and controls by using the χ^2^ test of proportions. We added variables that differed significantly (p<0.05) to univariate logistic regression to calculate ORs with 95% CIs and p values to assess the associations between the variables and HUS. We included the variables age, source of drinking water, and region of residence in stratified analysis to explore potential confounders and effect modifiers. We conducted multivariable logistic regression analysis (MVA) to control for negative and positive confounding and to calculate adjusted ORs (aORs). All p values correspond to a 2-tailed test. To reduce omitted-variable bias, we added predictor variables with a significance level of p*<*0.2 (rather than p*<*0.05) in the univariate analyses to the initial MVA model, an approach that is supported in the literature (*28*,*29*). We used forward stepwise techniques to identify variables suited or unsuited to the model and excluded variables on the basis of model efficiency, as indicated by the Akaike information criterion (AIC), in combination with other statistical tests. 

We used multiple models to explore potential gene dependencies, variance inflation factor to explore collinearity, and the Hosmer-Lemeshow test to assess goodness of fit. We noted no evidence of poor fitting; the χ^2^ statistic for the final model was 11.9, d.f. = 8, and p *=* 0.156. We deemed variables with a significance level of p*<*0.05 in the final MVA model to be independently associated with HUS.

### Ethics Approval

Formal consent was not required from patients in this study. STEC is a notifiable disease in Ireland, and formal consent is not required from patients to collect their data. CIDR data are collected as part of routine surveillance procedures, and laboratory testing records are collected as part of routine diagnostic and confirmatory testing. Approval was granted from the CIDR National Peer Review Committee to use CIDR data for the purposes of this study.

## Results

### Patient Demographic Data

Among 524 patients, 233 (44%) were 0–9 years of age and 291 (56%) were >10 years of age (Table 1); 53% were female and 47% male. The highest proportion of patients was in the South (20%; n = 104) followed by the Southeast and East (each 16%; n = 82). Ninety-three (18%) patients had outbreak-associated infection (Table 2).

**Table 1:** Timing of illness onset and demographic information for case-patients and controls in study of HUS among patients with STEC, Ireland, 2017–2020*

Category	HUS-STEC case-patients	Non–HUS-STEC controls	p value†	Total
Total	108	416		524
Year of STEC diagnosis				
2017	23 (21)	76 (18)	0.581	99 (19)
2018	34 (31)	118 (28)		152 (29)
2019	22 (20)	110 (26)		132 (25)
2020	29 (27)	112 (27)		141 (27)
Season of STEC diagnosis				
Autumn, August–October	53 (49)	160 (38)	<0.05	213 (41)
Winter, November–January	17 (16)	48 (12)		65 (12)
Spring, February–April	9 (8.3)	73 (18)		82 (16)
Summer, May–June	29 (27)	135 (32)		164 (31)
Patient age, y				
0–4	54 (50)	125 (30)	<0.05	179 (34)
5–9	23 (21)	31 (7.5)		54 (10)
10–14	8 (7.4)	34 (8.2)		42 (8.0)
15–64	9 (8.3)	150 (36)		159 (30)
>65	14 (13)	76 (18)		90 (17)
Age range, y				
0–9	77 (71)	156 (38)	<0.05	233 (44)
>10	31 (29)	260 (63)		291 (56)
Sex				
F	63 (58)	215 (52)	0.217	278 (53)
M	45 (42)	201 (48)		246 (47)
Region				
East	23 (21)	59 (14)	<0.05	82 (16)
Northeast	7 (6.5)	40 (9.6)		47 (9)
Midlands	7 (6.5)	32 (7.7)		39 (7.4)
Northwest	8 (7.4)	16 (3.8)		24 (4.6)
Midwest	19 (18)	63 (15)		82 (16)
West	10 (9.3)	54 (13)		64 (12)
South	26 (24)	78 (19)		104 (20)
Southeast	8 (7.4)	74 (18)		82 (16)
Resident of Ireland				
N	0	2 (0)	0.436	2 (0)
Y	69 (64)	227 (55)		296 (56)
Missing	39 (36)	187 (45)		226 (43)

*Values are no. (%) except as indicated. HUS, hemolytic uremic syndrome; STEC, Shiga toxin–producing *Escherichia coli*. †By χ^2^ test.

**Table 2:** Reported symptoms and risk factors for case-patients and controls in study of HUS among patients with STEC, Ireland, 2017–2020*

Category	HUS-STEC case-patients	Non–HUS-STEC controls	p value†	Total
Home in rural location				
N	24 (22)	72 (17)	0.073	96 (18)
Y	27 (25)	142 (34)		169 (32)
Missing	57 (53)	202 (49)		259 (49)
Reported vomiting				
N	21 (19)	250 (60)	<0.05	271 (52)
Y	83 (77)	131 (31)		214 (41)
Missing	4 (3.7)	35 (8.4)		39 (7.4)
Reported bloody diarrhea				
N	44 (41)	224 (54)	<0.05	268 (51)
Y	53 (49)	149 (36)		202 (39)
Missing	11 (10)	43 (10)		54 (10)
Outbreak associated				
N	68 (63)	363 (87)	<0.05	431 (82)
Y	40 (37)	53 (13)		93 (18)
Traveled abroad within past 10 d				
N	93 (86)	345 (83)	0.889	438 (84)
Y	8 (7.4)	28 (6.7)		36 (6.9)
Missing	7 (6.5)	43 (10)		50 (10)
Home drinking water				
Public	60 (56)	226 (54)	0.996	286 (55)
Private well	28 (26)	107 (26)		135 (26)
Group scheme, public supply	7 (6.5)	28 (6.7)		35 (6.7)
Group scheme, private	3 (2.8)	13 (3.1)		16 (3.1)
Missing	10 (9.3)	42 (10)		52 (10)
Consumed unpasteurized cheese or milk				
N	86 (80)	346 (83)	0.714	432 (82)
Y	4 (3.7)	13 (3.1)		17 (3.2)
Missing	18 (17)	57 (14)		75 (14)
Risk group				
Not in a risk group	62 (57)	292 (70)	<0.05	354 (68)
Child in crèche	29 (27)	59 (14)		88 (17)
Attends other institution	5 (4.6)	8 (1.9)		13 (2.5)
Childcare worker	1 (0.9)	14 (3.4)		15 (2.9)
Food handler	1 (0.9)	8 (1.9)		9 (1.7)
Missing	10 (9.3)	35 (8.4)		45 (8.6)
Recent outdoor recreational activities or recreational farmland contact‡				
N	71 (66)	289 (69)	<0.05	360 (69)
Y	26 (24)	61 (15)		87 (17)
Missing	11 (10)	66 (16)		77 (15)
Contact with farm animals				
No contact	56 (52)	209 (50)	<0.05	265 (51)
Regular contact§	26 (24)	122 (29)		148 (28)
One-off, e.g., visit to a pet farm	9 (8.3)	22 (5.3)		31 (5.9)
Regular and one-off contact	3 (2.8)	11 (2.6)		14 (2.7)
Contact of unknown nature	4 (3.7)	2 (0.5)		6 (1.1)
Missing	10 (9.3)	50 (12)		60 (11)

*Values are no. (%) except as indicated. HUS, hemolytic uremic syndrome; STEC, Shiga toxin–producing *Escherichia coli*. †By χ^2^ test. ‡Hillwalking, camping, swimming in lakes, water sports, or beach. §Lives, works, or is cared for on a farm, or exposure to livestock, manure, slurry, or sewage through household contacts.

### Patient Isolate Genomic Data

Overall, the *stx* subtypes most commonly found in patients’ isolates were *stx2a* alone (27%; n = 144), *stx1a* alone (20%; n = 105), or both *stx1a* and *stx2a* (29%; n = 154) (Table 3). The most common subtypes among case isolates were *stx2a* alone (52%; n = 56) or *stx1a* and *stx2a* (38%; n = 41). Four (4%) cases had *stx1a* alone. Isolates from 419 (80%) patients contained *eae* genes, wherein β1 (38%; n = 198) and γ1 (31%; n = 161) subtypes were predominant, similar to the 102 (94%) HUS cases, where γ1 (45%; n = 49) and β1 (38%; n = 41) were also predominant. Ninety-five (88%) case and 360 (87%) control isolates contained the *ehxA* gene. Isolates from 187 (36%) patients were serogroup O26, and isolates from 122 (23%) patients were serogroup O157.

**Table 3:** Distribution of virulence genes and serogroups for case-patients and controls in study of HUS among patients with STEC, Ireland, 2017–2020*

Category	HUS-STEC case-patients	Non–HUS-STEC controls	p value†	Total
*stx* genes‡				
*stx1a* alone	4 (3.7)	101 (24)	<0.05	105 (20)
*stx1a* + *stx2a*	41 (38)	113 (27)		154 (29)
*stx1a* + *stx2c*	3 (2.8)	25 (6)		28 (5.3)
*stx2a* alone	56 (52)	88 (21)		144 (27)
*stx2c* alone	1 (0.9)	7 (1.7)		8 (1.5)
Other combinations	1 (0.9)	79 (19)		80 (15)
Missing	2 (1.9)	3 (0.7)		5 (1.1)
*eae* genes present				
N	6 (5.6)	99 (24)	<0.05	105 (20)
Y	102 (94)	317 (76)		419 (80)
*eae* subtype				
None	6 (5.6)	99 (24)	<0.05	105 (20)
β1	41 (38)	157 (38)		198 (38)
γ1	49 (45)	112 (27)		161 (31)
ε1	4 (3.7)	19 (4.6)		23 (4.4)
ζ3	3 (2.8)	16 (3.8)		19 (3.6)
θ	4 (3.7)	10 (2.4)		14 (2.7)
κ	0	1 (0.2)		1 (0.2)
ξ	1 (0.9)	2 (0.5)		3 (0.6)
*ehxA* gene present				
N	5 (4.6)	41 (10)	0.106	46 (8.8)
Y	95 (88)	360 (87)		455 (87)
Missing	8 (7.4)	15 (3.6)		23 (4.4)
Serogroup				
O26	39 (36)	148 (36)	<0.05	187 (35.7)
O157	28 (26)	94 (23)		122 (23.3)
O145	15 (14)	16 (3.8)		31 (5.9)
O103	5 (4.6)	18 (4.3)		23 (4.4)
O55	6 (5.6)	5 (1.2)		11 (2.1)
O111	3 (2.8)	3 (0.7)		6 (1.1)
Other O group	11 (10)	130 (31)		141 (26.9)
Missing	1 (0.9)	2 (0.5)		3 (0.6)

*Values are no. (%) except as indicated. HUS, hemolytic uremic syndrome; STEC, Shiga toxin–producing *Escherichia coli*. †By χ^2^ test. ‡Composite variable; distribution by individual *stx* gene shown in Appendix 1.

### Genomewide Association Study on Microbial Genomic Factors Associated with HUS

The pangenome for the 531 STEC isolates contained 63,763 genes, from which 1,246 were defined as core genes present in 99% of the isolates. Twenty-six accessory genes had statistically significant associations with HUS (Table 4). Of those, 7 genes encoded hypothetical proteins with unknown function; the other 19 genes were functionally annotated and predicted to be involved in different processes, such as toxin production (*stx2B*), phage life cycle (*ybc*Q_1, *ydf*U_1), transcriptional regulation (group_5720, *yiaU*, *yedW*_10), transporters (*fieF*, *purP*, *sbp*), sugar (*pfkA*, *tpiA*) and lipid (*cdh*) metabolisms, detoxification (*sodA*, *yiiM*, *rsxG*), and stress response (*uspD*, *cpxA and cpxP*, *ygiW_2*).

**Table 4:** Genes with positive association with HUS after genomewide association analysis in study of HUS development among persons with STEC, Ireland, 2017–2020*

Gene annotation	Functional annotation	OR†
*stx2B*	Shiga toxin 2 subunit B	6.4
*ybcQ_1*	DLP12 prophage; predicted antitermination protein	5.4
group_31760	Hypothetical protein	4.4
*ydfU_1*	Qin prophage; predicted protein	4.3
group_30198	Hypothetical protein	4.4
group_31748	Hypothetical protein	4.7
group_30187	Hypothetical protein	5.0
group_5720/*mokC*_2‡	Regulatory peptide whose translation enables *hokC* (gef) expression	2.8
*sodA*	Superoxide dismutase (Mn)	2.6
*pfkA*	6-phosphofructokinase-1 monomer	2.6
group_33058	Hypothetical protein	2.5
*fieF*	Zn2+/Fe2+/Cd2+ efflux transporter FieF	2.5
group_36684	Hypothetical protein	2.5
*yiaU*	Putative DNA binding transcriptional regulator, LysR-type	4.6
*tpiA*	Triose phosphate isomerase monomer	2.5
*uspD*	Stress protein involved in resistance to UV irradiation	2.4
group_20906	Adenine:H+ symporter	2.4
group_31570	Hypothetical protein	2.4
*cpxP*	Regulator of the Cpx response and possible chaperone involved in resistance to extracytoplasmic stress	2.4
group_34824	Putative DNA binding response regulator in 2-component system with YedV	2.4
*sbp*	Sulfate/thiosulfate ABC transporter – periplasmic binding protein Sbp	2.4
*cpxA*	Sensor histidine kinase CpxA	2.4
*yiiM*	Protein involved in base analog detoxification	2.3
*cdh*	CDP-diglyceride hydrolase/CDP-diacylglycerol pyrophosphatase	2.3
*ygiW_2*	Stress-induced protein	2.6
*rsxG*	Member of SoxR-reducing complex	4.0

*N = 524 patients. HUS, hemolytic uremic syndrome; OR, odds ratio; STEC, Shiga toxin–producing *Escherichia coli*. †OR >1, Benjamini-Hochberg p<0.05, pairwise comparisons p<0.05. ‡Nonunique gene name where sequences with the same gene name have ended up in different groups. It might be because of split genes or misannotation.

### Results of Multivariable Analysis

We assessed 47 variables in the MVA, including the patient characteristics, epidemiologic factors, virulence genes, serogroups (Tables 5,6,7), and all 26 genes that had statistically significant associations in the GWAS (Appendix 2 Table 1). Variables in the ﬁnal MVA model were age, region, outbreak association, *stx* subtypes, *eae* subtypes, and *ehxA*, *pfkA*, *fieF*, *ygiW_2*, and group_5720. We observed potential dependencies or synergies between *ygiW_2* and group_5720 and between *pfkA* and *fieF* during development of the MVA model. To resolve that issue, we created 2 composite variables, *ygiW_2/*group_5720 and *pfkA/fieF*. Variables that we assessed but that did not remain in the final MVA model were season of STEC diagnosis; reported vomiting and reported bloody diarrhea; risk group (child in crèche, recent outdoor recreational activities or recreational farmland contact, contact of an unknown nature with farm animals or their feces); *eae* subtypes γ1, β1, and θ1; *ehxA*; and all genes from the GWAS except for *pfkA*, *fieF*, *ygiW_2*, and group_5720.

**Table 5:** Univariate and multivariable analysis of demographic factors associated with HUS development among persons with STEC, Ireland, 2017–2020*

Category	Unadjusted OR (95% CI)	p value†	Adjusted OR (95% CI)	p value†
Year of STEC diagnosis				
2017	Referent		NI	
2018	1 (0.5–1.8)	0.873		
2019	0.7 (0.3–1.3)	0.214		
2020	0.9 (0.5–1.6)	0.622		
Season of STEC diagnosis				
Autumn	Referent		NI	
Winter	1.1 (0.5–2)	0.836		
Spring	**0.4** (**0.2**–**0.8**)	**<0.05**		
Summer	**0.6** (**0.3**–**1.2**)	**0.152**		
Age, y				
0–4	**7.2** (**3.6**–**16.1**)	**<0.05**	NI	
5–9	**12.4** (**5.4**–**30.7**)	**<0.05**		
10–14	**3.9** (**1.4**–**11**)	**<0.05**		
15–64	Referent			
>65	**3.1** (**1.3**–**7.7**)	**<0.05**		
Age group, y				
0–9	**4.1** (**2.6**–**6.6**)	**<0.05**	**3.3 (1.8**–**6.4)**	**<0.05**
>10	Referent			
Sex				
F	Referent		NI	
M	0.8 (0.5–1.2)	0.218		
Region				
East	Referent			
Northeast	**0.4** (**0.2**–**1.1**)	**0.094**	**0.2 (0**–**0.6)**	**<0.05**
Midlands	**0.6** (**0.2**–**1.4**)	**0.233**	**0.2 (0.1**–**0.7)**	**<0.05**
Northwest	1.3 (0.5–3.3)	0.617	0.4 (0.1–1.4)	0.168
Midwest	0.8 (0.4–1.6)	0.475	**0.3 (0.1**–**0.7)**	**<0.05**
West	**0.5** (**0.2**–**1.1**)	**0.078**	**0.2 (0.1**–**0.7)**	**<0.05**
South	0.9 (0.4–1.7)	0.639	0.5 (0.2–1.3)	0.165
Southeast	**0.3** (**0.1**–**0.6**)	**<0.05**	**0.1 (0**–**0.4)**	**<0.05**
Resident of Ireland				
N	Referent		NI	
Y	1.75 × 10^8^	0.989		

*N = 524 patients. Bold indicates significance. HUS, hemolytic uremic syndrome; NI, not included in final model; OR, odds ratio; STEC, Shiga toxin–producing *Escherichia coli*. †By χ^2^ test.

**Table 6:** Univariate and multivariable analysis of epidemiologic and microbial genomic factors associated with HUS development among persons with STEC in Ireland, 2017–2020*

Category	Unadjusted OR (95% CI)	p value†	Adjusted OR (95% CI)	p value†
Home in rural location				
N	Referent		NI	
Y	0.6 (0.3–1.1)	0.075		
Reported vomiting				
N	Referent		NI	
Y	**7.5 (4.6–13)**	**<0.05**		
Reported bloody diarrhea				
N	Referent		NI	
Y	**1.8 (1.2–2.9)**	**<0.05**		
Outbreak associated				
N	Referent			
Y	**4 (2.5–6.6)**	**<0.05**	**3.55 (1.8–7.2)**	**<0.05**
Traveled abroad within past 10 d				
N	Referent		NI	
Y	1.1 (0.4–2.3)	0.889		
Home drinking water				
Public	Referent		NI	
Private well	1 (0.6–1.6)	0.955		
Group scheme, public supply	0.9 (0.4–2.2)	0.893		
Group scheme, private	0.9 (0.2–2.8)	0.831		
Consumed unpasteurized cheese or milk				
N	Referent		NI	
Y	1.2 (0.3–3.6)	0.715		
Risk group				
Not in a risk group	Referent		NI	
Child in crèche	**2.3** (**1.4**–**3.9**)	**<0.05**		
Attends other institution	2.9 (0.9–9.1)	0.066		
Childcare worker	0.3 (0–1.7)	0.297		
Food handler	0.6 (0–3.3)	0.62		
Recent outdoor recreational activities or recreational farmland contact‡				
N	Referent		NI	
Y	**1.7** (**1**–**2.9**)	**<0.05**		
Contact with farm animals or their feces				
No contact	Referent		NI	
Regular contact§	1 (0.5–1.3)	0.384		
One-off, e.g., visit to a pet farm	1.5 (0.6–3.4)	0.317		
Regular and one-off contact	1 (0.2–3.4)	0.979		
Contact of unknown nature	**7.5** (**1.4–54.8**)	**<0.05**		

*N = 524 patients. Bold indicates significance. HUS, hemolytic uremic syndrome; NI, not included in final model; OR, odds ratio; STEC, Shiga toxin–producing *Escherichia coli*. †By χ^2^ test. ‡Hillwalking, camping, swimming in lakes, water sports, beach. §Lives, works, or is cared for on a farm, or exposure to livestock, manure, slurry, or sewage through household contacts.

**Table 7:** Univariate and multivariable analysis of microbial genomic factors associated with HUS development among persons with STEC in Ireland, 2017–2020*

Category	Unadjusted OR (95% CI)	p value†	Adjusted OR (95% CI)	p value†
*stx* genes				
*stx1a* alone	Referent			
*stx1a* + *stx2a*	**9.2 (3.5–31.3)**	**<0.05**	**36.75 (7.4–358.4)**	**<0.05**
*stx1a* + *stx2c*	**3 (0.6–14.6)**	**0.164**	**31.37 (2.9–447.4)**	**<0.05**
*stx2a* alone	**16 (6.3–54.6)**	**<0.05**	**154 (27.2–1,567)**	**<0.05**
*stx2c* alone	3.6 (0.2–29)	0.279	9.88 (0.3–206.5)	0.139
Other *stx1* and *stx2* combinations	0.3 (0–2.2)	0.312	2.78 (0.1–58.5)	0.507
*eae* genes				
N			NI	
Y	**5.3 (2–13.9)**	**<0.05**		
*eae* subtype				
None	Referent			
β1	**4.3 (2–11.6)**	**<0.05**	0.4 (0.1–2.8)	0.345
γ1	**7.2 (3–19.5)**	**<0.05**	0.25 (0–1.4)	0.097
ε1	3.5 (1–13.4)	0.072	1.47 (0.1–18)	0.755
ζ3	3.1 (1–13)	0.136	5.82 (0.2–109.2)	0.244
θ	**6.6** (**1–27.4**)	**<0.05**	1.43 (0.1–14.5)	0.764
κ	0 (NC–4.80 x 10^42^)	0.098	NI	
ξ	8.3 (0–99.4)	0.103	0.57 (0–26.3)	0.795
*ehxA*				
N	Referent			
Y	**1 (1–6.4)**	**0.113**	0.39 (0.1–1.8)	0.215
*pfk*A*/fieF*				
Neither *pfkA* nor *fieF*	Referent			
Both *pfkA* and *fieF*	**2 (1–3.1)**	**<0.05**	**1.82 (1–3.4)**	**0.052**
*ygiW_2/*group 5720				
Neither *ygiW_2* nor group_5720	Referent			
Both *ygiW_2* and group_5720	**3.9 (2.2–7.6)**	**<0.05**	**5.49 (1.9–18.6)**	**<0.05**
Group_5720 only	2 (0.1–13.5)	0.55	2.92 (0.1–44.9)	0.471
*ygiW_2* only	1.6 (0.7–3.7)	0.238	2.58 (0.8–9.3)	0.134
Serogroup				
O26	**3.1 (1.6–6.6)**	**<0.05**	NI	
O157	**3.5 (1.7–7.7)**	**<0.05**		
O145	**11.1 (4.4–29)**	**<0.05**		
O103	**3.3 (0.9–10.2)**	**<0.05**		
O55	**14.2 (3.7–57)**	**<0.05**		
O111	**11.8 (2–71)**	**<0.05**		
Other O group	Referent			

*N = 524 patients. Bold indicates significance. HUS, hemolytic uremic syndrome; NC, not calculable; NI, not included in final model; OR, odds ratio; STEC, Shiga toxin–producing *Escherichia coli*. †By χ^2^ test.

In MVA, younger patients (0–9 years of age) had 3-fold odds of HUS compared with those >10 years of age (aOR 3.3 [95% CI 1.7–6.4]). Patients residing in regions other than the East had lower odds of developing HUS compared with those resident in the East (Northeast aOR 0.2 [95% CI 0.0–0.6], Midlands aOR 0.2 [95% CI 0.1–0.7], Midwest aOR 0.3 [95% CI 0.1–0.7], West aOR 0.2 [95% CI 0.1–0.7], and Southeast aOR 0.1 [95% CI 0.0–0.4]). Persons with outbreak-associated infection had >3-fold odds of HUS compared with persons whose infection was not outbreak-associated (aOR 3.5 [95% CI 1.8–7.2]). Compared with patients who had *stx1a* alone, the odds of HUS were higher among patients with *stx2a* alone (aOR 154.3 [95% CI 27.1–1,567.3]), both *stx1a* and *stx2a* (aOR 36.7 [95% CI 7.3–358.4]), or both *stx1a* and *stx2c* (aOR 31.3 [95% CI 2.9–447.4]).

The inclusion of the genes *ygiW_2* (aOR 3.2 [95% CI 1.2–9.1]) or group_5720 (aOR 2.6 [95% CI 1.3–5.3]) had positive associations with HUS in forward stepwise regression, but only group_5720 remained statistically significant when we made attempts to incorporate both genes as independent variables. A combined *ygiW_2*/group_5720 variable had increased odds (aOR 5.4 [95% CI 1.8–18.6]) and provided a better model fit.

Similarly, when assessed independently, the inclusion of *pfkA* (aOR 2.0 [95% CI 1.1–2.7]) showed a positive association with HUS, but *fieF* (aOR 0.03 [95% CI 0.0–0.92]) showed a negative association and a considerable increase in odds for *pfkA* was seen when *fieF* was added to the model (aOR 58.05 [95% CI 1.9–1,104.7]). A combined *pfkA*/*fieF* variable had an overall positive association (aOR 1.8 [95% CI 1.0–3.3]) and provided a better model fit.

### Phylogeny of HUS and Non-HUS STEC Isolates

HUS cases were distributed across several serogroups. Those serogroups were O26 (36%), O157 (26%), O145 (14%), O103 (4.6%), O111 (2.8%), and O55 (5.6%) (Figure).

## Discussion

Consistent with the findings of previous studies, we found that young age, outbreak-associated infection, and region of residence in Ireland were associated with HUS developing in STEC patients during the study period (*4*,*30*–*32*). The higher odds of HUS among patients residing in the East of Ireland (likely representing a more urban environment) might be because patients in more rural environments are protected by repeated previous STEC exposures, although we cannot confirm that hypothesis. Another possible reason is the higher density of childcare facilities in the East region; children are more likely to be associated with an STEC outbreak in a childcare setting in the East and therefore may have a higher risk for HUS. Being part of an STEC outbreak was associated with HUS, possibly because of increased virulence of pathogenic strains linked to outbreaks. Other factors that were associated with HUS in previous studies were season of infection and having reported bloody diarrhea and vomiting (*4*,*30*–*32*), factors that were significant in our univariate analysis but not in our MVA. Even though bloody diarrhea and vomiting were not significant, it is arguable that in the absence of information on symptom onset date, as in our study, those factors should not be included because of potential for causal confounding.

Also consistent with the findings of previous studies, we found that the presence of *stx2* genes was independently associated with HUS (*4*,*33*). We demonstrated that the subtype *stx2a* alone had a stronger association with HUS compared with presence of *stx1a* alone or *stx1* and *stx2* subtype combinations (*34*). We further found that the combined presence of *stx1a* and *stx2a* was independently associated with HUS.

The presence of *eae* genes, described elsewhere as being associated with HUS (*5*,*11*,*35*–*37*), was not significantly associated with HUS in our study. That difference may be because of the collinearity we observed between *stx* and *eae*. Other genes involved in adherence, such as *tir*, *toxB*, and the *sfp* and *lpf* gene clusters, were not associated with HUS in our study (*38*). We excluded serogroup from MVA because of known collinearity with *stx* genes. The non–locus of enterocyte effacement–encoded immune system modulator *nle*H1–2 has been reported to be associated with HUS (*30*) but was not identified in our GWAS.

The application of GWAS methodology to public health research on STEC infections is relatively uncommon. STEC GWAS studies in other countries have focused on different outcomes (e.g., bloody diarrhea) or have been limited in sample size (*34*,*37*). Using GWAS, we identified 26 putative genes that were significantly associated with HUS but whose definitive role in HUS pathogenesis remains to be elucidated. Functional annotation suggests their involvement in processes such as toxin production, phage life cycle, transcriptional regulation, transporters, and stress response.

Only the 2 composite gene pairs *pfkA/fieF* and *ygiW_2/*group_5720 were significantly associated with HUS in MVA. Of note, *pfkA* and *fieF* are contiguous in the genome and have the same presence/absence pattern, supporting the theory of gene dependency. The *fieF* gene was negatively associated with HUS when added to the model as an independent variable, but when coupled with *pfkA*, it was positively associated and improved the model fit. Information on the potential role of those genes is limited. The *pfkA* gene product is a phosphofructokinase and a key component in the glycolytic pathway, enabling *E. coli* to utilize glucose as a carbon source (*39*), whereas the *fieF* gene encodes an iron/zinc/cadmium efflux transporter that forms part of a detoxification mechanism (*40*–*42*). Previous studies describe a role for *ygiW* in tolerance to cadmium, oxidative stress (*43*,*44*), and biofilm growth (*45*), whereas the group_5720 gene product appears to be similar to *mokC* (through functional annotation), a mediator in plasmid stabilization (*46*). Further research is warranted to explore how those genes could be interacting, and how they modulate STEC virulence and potentially contribute to HUS development.

One strength of our study is that we used data from a full national cohort of notified infections, minimizing potential bias where possible through study design. In contrast to prior studies on STEC-associated HUS, we had a large number of HUS cases (*5*,*11*,*30*) and used complete molecular data from the national strain collection.

For the novel gene associations, our findings should be interpreted cautiously. The ORs for gene pairs *ygiW_2*/group_5720 and *pfkA*/*fieF* were modest, and the role of those genes in pathogenesis needs to be further elucidated. For example, we did not measure the level of gene expression and regulation, which plays a fundamental role in virulence. We did explore potential gene interdependencies and interactions by using forward and backward stepwise regression techniques, but even though we identified interactions between 2 gene pairs, more may exist.

Regarding limitations of our study, we took measures to mitigate potential biases resulting from screening policies for STEC outbreaks in Ireland. Unknown biases might have resulted from exclusion of patients that did not yield culture-positive isolates and either could not be linked to a laboratory record or were linked but did not have associated isolate genomes. Whereas s*tx2* is more often associated with high-risk STEC isolates, isolates for 4 (4%) HUS cases were detected with s*tx1* only, even though we made every effort to find a co-infecting *stx2*-producing strain through exhaustive accredited laboratory methods. We cannot exclude the possibility that a co-infecting *stx2*-producing STEC was present at some point between illness onset date and sample collection, which ranged up to several weeks, and was not detectable in the sample. Recall bias was not possible in MVA, since the variables included were based on factual information. The R^2^ value of the MVA model suggested that 35% of the outcome (HUS) could be explained by the independent variables, indicating that other factors influence HUS development. Relevant data on volume of drinking water, underlying medical conditions, and other host factors; clinical management, including antimicrobial drug treatment; and longer-term data on outcomes were not available because those data are not collected in routine STEC surveillance in Ireland. In addition, variables of interest collected in routine surveillance, including recent outdoor recreational activities or recreational farmland contact, contact with farm animals or their feces, and residence in an urban or rural location, had a high number of missing observations, reducing the precision of results. We instead determined residence distribution on the basis of the administrative region. Furthermore, incomplete data for other variables may have negatively impacted their suitability to MVA. The type of GWAS carried out in this study also has limitations, assessing only presence or absence of accessory genes, omitting important genetic variation caused by single-nucleotide polymorphisms and insertions or deletions that could be explored through other GWAS methodologies (*47*–*50*).

In conclusion, this study benefitted from the use of a full national cohort of notified infections with complete molecular data and is another step toward clarifying the factors influencing HUS development among STEC patients. The roles of genes and their dependencies and synergies in STEC pathogenesis should be further investigated, particularly the role of the novel genes identified using GWAS. Our findings, particularly if validated by further studies, could improve early identification of higher-risk STEC infection and help guide enhanced surveillance and public health management.

Genome assemblies of STEC isolates analyzed in this study are accessible in GenBank (BioProject nos. PRJNA1096451 and PRJNA1096304).

Appendix 1 Isolate data for study of hemolytic uremic syndrome among Shiga toxin–producing *Escherichia coli* patients, Ireland, 2017–2020.

Appendix 2 Additional information for study of hemolytic uremic syndrome among Shiga toxin–producing *Escherichia coli* patients, Ireland, 2017–2020.
