# Supplementary material for: Case–Control Study of Factors Associated with Hemolytic Uremic Syndrome among Shiga Toxin–Producing Escherichia coli Patients, Ireland, 2017–2020
Source: Emerg Infect Dis. 2025 Apr;31(4):728–40. doi: 10.3201/eid3104.240060 (PMC11950266; doi:10.3201/eid3104.240060)
Supplement: Appendix 2 — Additional information for study of hemolytic uremic syndrome among Shiga toxin–producing Escherichia coli patients, Ireland, 2017–2020. [file 24-0060-Techapp-s2.pdf]

Article DOI: https://doi.org/10.3201/eid3104.240060

EID cannot ensure accessibility for supplementary materials supplied by authors. Readers who have difficulty accessing supplementary content should contact the authors for assistance.

Case–Control Study of Factors Associated with Hemolytic Uremic Syndrome among Shiga Toxin–Producing *Escherichia coli* Patients, Ireland, 2017–2020

Appendix 2

Appendix 2 Table 1. Microbial genomic factors (individual *stx* genes) associated with hemolytic uremic syndrome development among people notified with Shiga toxin-producing *Escherichia coli* in Ireland, case-control study 2017–2020

		Cases (HUS)		Controls (Non-HUS)						
		n	%	n	%					
Total	Genes	108		416		OR	95% CI			p value*
stx1	None	58	54	116	28	Ref				
	stx1a	48	44	246	59	**0.4**	**0.25**	**-**	**0.61**	**<0.05**
	stx1c	0	0.0	48	12	0.0	NA	-	NA	0.976
	stx1d	0	0.0	3	0.7	0.0	NA	-	NA	0.994
	Missing	2	1.9	3	0.7					
stx2†	None	4	3.7	133	32	Ref				
	stx2a†	97	90	201	48	**16.0**	**6.5**	**-**	**53**	**<0.05**
	stx2b	1	0.9	37	8.9	0.9	0.0	-	6.3	0.925
	stx2c†	4	3.7	32	7.7	4.2	0.9	-	18	0.052
	stx2d	0	0.0	6	1.4	0.0	NA	-	NA	0.989
	stx2e	0	0.0	4	1.0	0.0	NA	-	NA	0.991
	Missing	2	1.9	3	0.7					

*By χ^2 test. Bold text indicates statistical significance.
†One control had both *stx2a* and *stx2c*, and one control had both *stx2c* and *stx2a*: for both controls the second *stx* gene was excluded from the analysis.

Appendix 2 Table 2. Microbial genomic factors from GWAS (n = 26 genes) associated with hemolytic uremic syndrome development among people notified with Shiga toxin-producing *Escherichia coli* in Ireland, case-control study 2017–2020

		Cases (HUS)		Controls (Non-HUS)						
		n	%	n	%					
Total	Gene present	108	-	416	-	OR	95% CI			p value*
stx2B	No	12	11	183	44	Ref				
	Yes	96	89	233	56	**6.3**	**3.5**	**-**	**12**	**<0.05**
ybcQ_1	No	18	17	212	51	Ref				
	Yes	90	83	204	49	**5.2**	**3.1**	**-**	**9.2**	**<0.05**
ydfU_1	No	24	22	227	55	Ref				
	Yes	84	78	189	45	**4.2**	**2.6**	**-**	**7.0**	**<0.05**
group_30198	No	22	20	217	52	Ref				
	Yes	86	80	199	48	**4.3**	**2.6**	**-**	**7.2**	**<0.05**
group_31760	No	22	20	218	52	Ref				
	Yes	86	80	198	48	**4.3**	**2.6**	**-**	**7.3**	**<0.05**
rsxG	No	6	5.6	80	19	Ref				
	Yes	102	94	336	81	**4.1**	**1.9**	**-**	**11**	**<0.05**
sodA	No	32	30	219	53	Ref				
	Yes	76	70	197	47	**2.6**	**1.7**	**-**	**4**	**<0.05**
pfkA	No	32	30	218	52	Ref				
	Yes	76	70	198	48	**2.6**	**1.7**	**-**	**4.2**	**<0.05**

		Cases (HUS)		Controls (Non-HUS)						
		n	%	n	%					
Total	Gene present	108	-	416	-	OR	95% CI			p value*
sbp	No	32	30	210	50	Ref				
	Yes	76	70	206	50	**2.4**	**1.6**	**-**	**3.9**	**<0.05**
cdh	No	32	30	208	50	Ref				
	Yes	76	70	208	50	**2.4**	**1.5**	**-**	**3.8**	**<0.05**
tpiA	No	31	29	209	50	Ref				
	Yes	77	71	207	50	**2.5**	**1.6**	**-**	**4.0**	**<0.05**
group_36684	No	31	29	210	50	Ref				
	Yes	77	71	206	50	**2.5**	**1.6**	**-**	**4.1**	**<0.05**
group_31570	No	31	29	207	50	Ref				
	Yes	77	71	209	50	**2.5**	**1.6**	**-**	**3.9**	**<0.05**
group_33058	No	30	28	208	50	Ref				
	Yes	78	72	208	50	**2.6**	**1.7**	**-**	**4.2**	**<0.05**
uspD	No	31	29	208	50	Ref				
	Yes	77	71	208	50	**2.5**	**1.6**	**-**	**4.0**	**<0.05**
fieF	No	33	31	219	53	Ref				
	Yes	75	69	197	47	**2.5**	**1.6**	**-**	**4.0**	**<0.05**
cpxP	No	31	29	208	50	Ref				
	Yes	77	71	208	50	**2.5**	**1.6**	**-**	**4.0**	**<0.05**
group_34824	No	31	29	208	50	Ref				
	Yes	77	71	208	50	**2.5**	**1.6**	**-**	**4.0**	**<0.05**
cpxA	No	31	29	206	50	Ref				
	Yes	77	71	210	50	**2.4**	**1.6**	**-**	**3.9**	**<0.05**
yiiM	No	31	29	205	49	Ref				
	Yes	77	71	211	51	**2.4**	**1.5**	**-**	**3.9**	**<0.05**
ygiW_2	No	17	16	141	34	Ref				
	Yes	91	84	275	66	**2.7**	**1.6**	**-**	**4.9**	**<0.05**
group_5720	No	32	30	223	54	Ref				
	Yes	76	70	193	46	**2.7**	**1.8**	**-**	**4.4**	**<0.05**
group_30187	No	8	7.4	117	28	Ref				
	Yes	100	93	299	72	**4.9**	**2.5**	**-**	**11**	**<0.05**
group_31748	No	10	9.3	132	32	Ref				
	Yes	98	91	284	68	**4.6**	**2.4**	**-**	**9.6**	**<0.05**
group_20906	No	60	56	313	75	Ref				
	Yes	48	44	103	25	**2.4**	**1.6**	**-**	**3.8**	**<0.05**
yiaU	No	6	5.6	86	21	Ref				
	Yes	102	94	330	79	**4.4**	**2.0**	**-**	**12**	**<0.05**

*By χ^2 test. Bold text indicates statistical significance.
